# A Causal View of the Role and Potential Limitations of Capitation in Promoting Whole Health System Performance

**DOI:** 10.3390/ijerph20054581

**Published:** 2023-03-04

**Authors:** David Bruce Matchar, Wei Xuan Lai, Ashish Kumar, John Pastor Ansah, Yeuk Fan Ng

**Affiliations:** 1 Program in Health Services and Systems Research, Duke-NUS Medical School, Singapore 169857, Singapore; 2 Department of Medicine, Duke University, Durham, NC 27708, USA; 3 Center for Community Health Integration, Case Western Reserve University, Cleveland, OH 44106, USA; 4 Yishun Health, Singapore 768828, Singapore

**Keywords:** capitation, casual loop diagram, value-based health, fee for service, health service transformation, health service innovation

## Abstract

For several decades, health systems in developed countries have faced rapidly rising healthcare costs without concomitant improvements in health outcomes. Fee for service (FFS) reimbursement mechanisms (RMs), where health systems are paid based on volume, contribute to this trend. In Singapore, the public health service is trying to curb rising healthcare costs by transitioning from a volume-based RM to a capitated payment for a population within a geographical catchment area. To provide insight into the implications of this transition, we developed a causal loop diagram (CLD) to represent a causal hypothesis of the complex relationship between RM and health system performance. The CLD was developed with input from government policymakers, healthcare institution administrators, and healthcare providers. This work highlights that the causal relationships between government, provider organizations, and physicians involve numerous feedback loops that drive the mix of health services. The CLD clarifies that a FFS RM incentivizes high margin services irrespective of their health benefits. While capitation has the potential to mitigate this reinforcing phenomenon, it is not sufficient to promote service value. This suggests the need to establish robust mechanisms to govern common pool resources while minimizing adverse secondary effects.

## 1. Background and Objectives

For several decades, healthcare costs have risen disproportionally to general costs [1,2,3]. In part, this cost inflation can be attributed to an ageing population with greater health service needs, resulting in an increase in price and intensity of services [4]. As costs have risen, one would hope that this would be justified by better population health. However, evidence suggests that increased spending is only weakly associated with improved outcomes [5,6,7].

One explanation for increased costs without comparable improvements in health outcomes is that historically the dominant payment structure in many countries has been and continues to be “fee for service” (FFS). Under FFS, reimbursement is based on an amount paid per unit of service provided, thus incentivizing greater service volume [8].

FFS reimbursement would be fine if high margin services (where reimbursements to providers for providing health services far outweigh the cost of providing those services) were necessarily high value (low additional cost of the service compared to alternative services relative to the additional health benefits (i.e., incremental cost-effectiveness)). However, FFS reinforces the provision of high-margin services irrespective of value [8,9]. Some services can command a high margin because they are particularly attractive to patients, such as those involving potentially curative procedures. Others have high margins because the provider is especially efficient in providing the service—such as laboratory tests done in a joint lab with the advantage of economies of scale. Because basic FFS itself does involve linking payment to value, some high-margin services are of low value. Take, for example, back surgery for lumbar spinal stenosis. From 2002 to 2007, there was a 15-fold increase in the rate of more complex back surgeries in the US. However, these complex procedures were associated with a nearly three-fold increase in the odds of life-threatening complications [10].

The most common “fixes” to alleviating cost inflation while improving health outcomes under FFS are crude system structure changes in the forms of administrative barriers (i.e., making calls and filling out forms) and provider exclusion. Often, administrative barriers go beyond requiring extra steps (e.g., innocuous but time-consuming calls and forms) to mandating pre-approval of high-cost services by a physician, or more often by a non-provider who may be guided by a list of “appropriateness” criteria [11]. Exclusion of providers involves identifying and eliminating from the “preferred provider list” those who tend to follow higher-than-average cost service patterns [12].

Much has been written on how these crude structural changes intended to contain cost inflation have secondary effects that diminish broader system performance—the so-called quadruple aim of high population health, sustainable per capita cost, patient satisfaction, and provider satisfaction [13,14]. The commonly used “fixes” to FFS may contain costs, but at the expense of high administrative overhead that can reduce population health by denying high-value services simply because they are high cost, and that promotes dissatisfaction of patients and providers caught in a bureaucratic morass. Nevertheless, what is clear is that accomplishing the herculean task of achieving the quadruple aim must involve changing the reimbursement mechanisms (RMs).

## 2. Alternatives to FFS

A RM alternative to FFS is capitation. In theory, under capitation, an entity is assigned responsibility for the health care of a defined population and for the distribution of resources to providers to deliver that care. This accountable entity is given a fixed amount of money for the care of the defined population, based on a formula intended to estimate what is enough to provide needed health services (i.e., a capitation pool), irrespective of how many or which specific services are delivered.

In practice, RMs take many possible forms [15,16,17,18,19,20,21,22,23,24]. RMs can be classified based on whether the rate of payment is set prospectively or retrospectively, whether the payment is made prospectively or retrospectively, and whether the rate of payment is based on a unit of input or a unit of outcome. Under capitation, the rate of payment is set prospectively, the payment is made prospectively, and the payment is based on a unit of outcome. That is, providers seek payment for rendered services from the accountable organization, and payments are distributed to providers from a capitation pool. Variations of this RM often involve cost sharing; if there is money left over at the end of the year, the organization and providers share the profit, and if there is a deficit, they share the loss. Different formulas for cost sharing are used by different organizations, with more or less of the difference between capitation payments and expenditures being attached to the provider. Other variations of capitation include capitation in conjunction with payments for meeting condition performance standards (Pay-for-Performance (P4P)), volume of services, as well as additional payments for innovation or education.

Capitation is attractive to payers as it provides more direct control of costs. In addition, capitation is advocated as a means of encouraging efficiency through a reduction in service production costs [25,26]. However, it can have unintended effects such as reduced quality of care due to reduced availability of potentially valuable services [27,28]. Capitation also may not eliminate low-value services. Even in simple capitation, “shiny new” services that have low health benefits may also be encouraged if they serve to enhance the prestige of the organization and increase the number of enrolled members with lower health needs relative to those implied by the capitation formula. Additionally, since the common form of what is termed as capitation is a hybrid with features of FFS, there is potential for some providers to continue to offer a high volume of high margin services, even if those services are of low value, particularly if those providers do not perceive a significant downside from cost sharing.

Singapore, among other countries, is in the process of adopting capitation in some form for services provided in the public sector. As noted above, the RHs have been funded based on a retrospective assessment of services provided (i.e., service volume), and under this RM, Singapore has achieved good health outcomes while containing costs. However, it now faces a set of stressors to the status quo, including one of the most rapidly aging populations in the world and increasing service needs [29]. What is notable about Singapore is that, due to its relatively small size, dominance of the public sector, and long-term governance perspective, it is aiming for a RM nationally that can improve overall system performance beyond solely managing costs. While distinctive in many ways, understanding the implications of various RMs as they are implemented internationally can inform the appropriate RM design for Singapore.

A regime change in a health system’s RM is a profound intervention with a complex set of design issues and a host of potential intended and unintended effects. In all health systems contemplating such a change, including Singapore, implementing a new RM that improves system performance must proceed without damaging structures and relationships (i.e., if one must repair a plane in flight, the repair should not cause the plane to crash). In Singapore, this includes retaining the existing framework for health system financing, which has served the country well [30].

Given the complexity of this task, we aim to develop an understanding of the causes of sub-optimal health system performance in a format that clarifies for a broad audience the causes of poor performance and potential solutions to achieve a successful transition.

The objective of this paper is to describe the development of a causal diagram to support this effort by providing an accessible visual representation of the relationship between RM and system outcomes. It begins with key features of a FFS-based health system to provide insight into how moving from a volume-based to a population-based RM is likely to influence the mix of high- and low-health benefit services.

Thus, this paper highlights features of RM innovation that may result in failure to achieve more satisfactory system performance for those engaged in transforming RM and encourages them to consider strategies to alleviate undesirable secondary effects. Using the CLD, stakeholders involved in transforming RM in Singapore can simulate alternative RMs through thought experiments to anticipate the effects of such a major policy change.

## 3. Methodology

We developed a Causal Loop Diagram (CLD) to visually represent the dynamic relationship between RM and basic metrics of system performance [31,32]. As tools to promote systems thinking, CLDs have the potential for enhancing shared understanding of complex problems [33,34,35], which in turn can promote transparency, participation, and the capacity building necessary to guide the development of appropriate governance of health systems [36]. CLDs have been used to assess linkages between RMs, service supply, and incentives in a case based and FFS scenario [37] and study the effectiveness of P4P programs [38], supporting the intuition that CLDs can be useful in thinking about the role of RMs in the health system.

We developed our CLD iteratively through interactions and informal discussions with more than 20 informants, both in the public sector of Singapore’s health system and as global experts in health system administration.

First, we met individually with senior administrators in the Singapore public sector health system to consult their views on the potential implications of capitation on system performance. Based on a consensus that the quadruple aim framework captured the key elements defining health system performance in principle [14], we elicited discussion of the role of the government, provider institutions, and healthcare providers in producing these four outcomes in the context of the public healthcare system in Singapore. For the exercise, private primary care was assumed to be seamlessly connected (e.g., via appropriate contracts) to the public sector for individuals who were users of public sector services.

Second, the research team, consisting of individuals with clinical and administrative experience, applied the causal statements from the stakeholders to develop a “seed CLD”, working backwards from the system performance outcomes, taking the inputs of the first stage into account, and progressively adding entities related to three key subsystems: government, provider institutions, and healthcare providers (e.g., physicians).

As a final step, the team again met with the stakeholders individually to critique the resulting CLD. The critiquing process was based on the framework of categories of legitimate reservation [39], in which the stakeholder can, for example, question entity existence, whether a causal relation exists or if an intermediate causal link must first be formed, and whether there was causal sufficiency (e.g., another factor must be present for a particular cause to result in the diagrammed effect). The CLD was modified as indicated, and the result is presented here.

For clarity, each entity in the CLD is numbered and referenced in text with a hash (#), and each cause/effect arrow is associated with a polarity: positive (+) when an increase in the entity at the arrow base leads to an increase in the entity at the arrowhead, and negative (−) when the opposite is true. Feedback loops are identified when some dynamics of the system reinforce or balance some aspect of system behavior. Such loops are denoted as reinforcing (with a notation of Rn) if the resulting feedback tends to promote growth of effects, and balancing (with a notation of Bn) if the feedback tends to promote inhibition of effects.

## 4. Results

### 4.1. The Causal Loop Diagram (CLD)

For clarity, the CLD developed by the team and reviewed by stakeholders is presented below in a series of four figures in which the CLD is built up in sections. Firstly, we describe the dynamics between population health and the way the health system meets health needs with services to improve health. Secondly, we describe how healthcare organizations influence the mix of services by adjusting physician rewards. Thirdly, we describe other factors that influence the mix of services provided by healthcare organizations. Lastly, we describe how the performance of the health system generates pressure for innovation by healthcare organizations.

**Figure 1:**
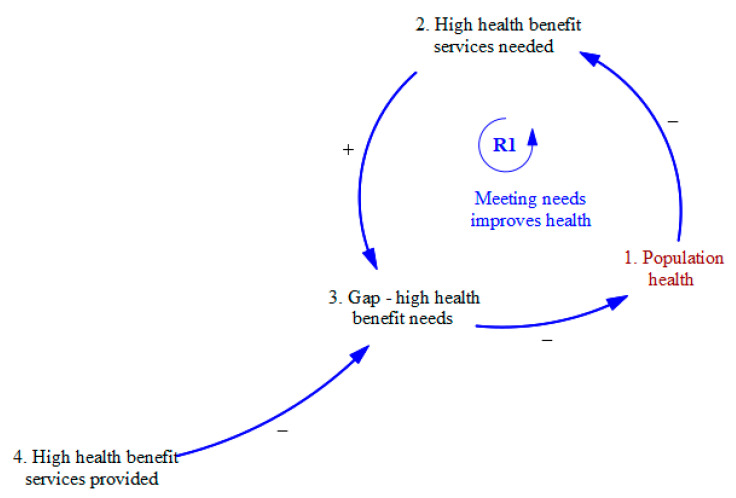
Meeting needs (with High Health Benefit Services) improves population health.

Figure 1 shows that the impact of the health system on population health is directly related to the degree to which the health system provides high-benefit services when such services are needed. For simplicity, health services needs are categorized into two types: low health benefit services (LHBS) and high health benefit services (HHBS). The benefit of a service when applied to a need is defined as the likelihood the service will reduce the rate of progression to a worse health state, avoidable use of acute services such as the emergency department or acute inpatient hospital, chronic institutionalization, or premature death [40], which when applied consistently would also improve aggregate measures of population health.

LHBS are those that, under many plausible indications, have limited or no impact on the listed measures of health. By way of illustration, LHBS include back surgery for acute low back pain in the absence of a neurological deficit [41,42] and angiography for individuals with a low probability of cardiac chest pain [43]. HHBS include physiotherapy post-stroke [44], cognitive behavior therapy for chronic pain [45], and palliative care services for individuals with limited life expectancy and physical or social/psychological distress [46].

As shown by the reinforcing feedback loop R1 in Figure 1, the provision of HHBS (#2) decreases the unmet high-benefit service’s needs (i.e., the gap in HHBS needed, #3). As fewer unmet high-benefit service needs, by definition, improve population health (#1), the result is a virtuous cycle—less need for HHBS (#2) as well as a reduction in the gap between HHBSs needed (#3) and those provided (#4). The stakeholders agreed that the provider organization will always want to influence the service mix to increase profitability (or, for a not-for-profit organization, to create surpluses that offer the opportunity to grow, recruit the most talented staff, and achieve economies of scale).

Under FFS, the objective of increased profit/surplus creates an incentive for high margin services, irrespective of their health benefits. However, under simple capitation, where payments to the organization are based purely on the number of individuals covered under the capitation arrangement, the organization goal is the same, but the principal incentive induced by the RM is to minimize high-cost service utilization and to drive down the cost of those services through increased efficiency (in terms of cost per unit of service, not cost per unit of health benefit).

In this simple representation, neither FFS nor simple capitation would lead the organization to especially want to influence the mix of services based on value, particularly if the population is rapidly turning over, as HHBS may only achieve cost savings in the long-term. However, if the healthcare needs of the population do not increase rapidly over time, HHBS may be of less interest to organizations under a FFS RM. Under FFS, organizations are reimbursed based on services provided. Since HHBS reduce future service needs, organizations are less incentivized to provide more HHBS.

For a health system that cares for a population over their life span, as with Singapore, profit/surplus considerations under capitation will make HHBS more attractive to the organization insofar as they lead to reduced future service needs as these future needs would come out of the future capitation pool (i.e., reduce future surplus). This would not be the case for capitated organizations, for which patient turnover is frequent and thus immediate costs are salient but future costs are another organization’s concern.

**Figure 2:**
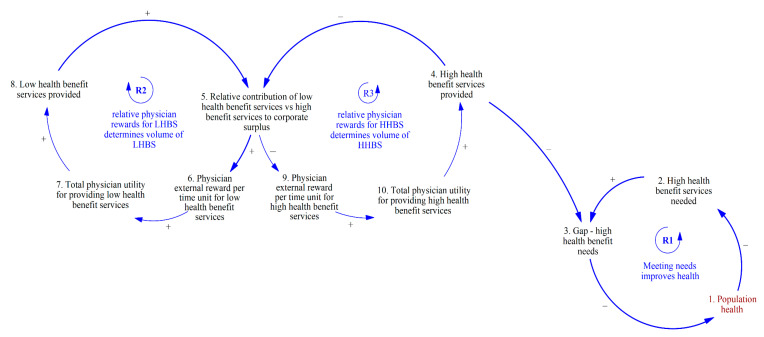
Competing dynamics for the provision of LHBS and HHBS within a corporation.

These considerations led to Figure 2: whatever the organizational priority under a RM, the primary consideration to determine the mix of HHBS and LHBS is the contribution of these respective services to corporate surplus (#5). The mechanism through which corporations influence this mix is by determining the physician reward per unit time for the LHBS and HHBS. Rewards per unit time can be tangible, through immediate increases in pay, higher future income via career advancement within the public sector, or monetary equivalents such as space, equipment, staff, and fellow training slots. Additionally, physicians may be more receptive to offering a mix of services that are desirable at the system level if they plan to move into the private (fee for service) sector, and time spent in the public sector increases their desirability as a private sector physician. Reward per unit time can also be intangible, such as gratification from performing HHBS rather than LHBS and public recognition for behaviors consistent with better system performance.

The reinforcing feedback loops R2 and R3 in Figure 2 show the causal structure of the retrospective volume-based payment-driven RMs on health services provided. The dynamics between R2 and R3 highlight the competition within a corporation for the provision of LHBS and HHBS, given that the capacity for providing is finite and must be proportioned between LHBS and HHBS provided. In R2, an increase in the relative contribution of LHBS versus HHBS to corporate surplus (#5) results in an increase in the external reward to the physician per unit time for LHBS (#6). Physicians therefore experience an increase in total utility in providing LHBS (#7), which results in a higher volume of LHBS provided (#8), thereby further increasing the relative contribution of LHBS to corporate surplus (#5). In R3, an increase in the relative contribution of LHBS to corporate surplus incentivizes the corporation to decrease the physician reward per unit time for providing LHBS (#9). Physicians would therefore experience a decrease in total utility in providing LHBS (#11) and decrease the volume of HHBS provided (#4), thereby further decreasing the relative contribution of LHBS to corporate surplus (#5). A decrease in the relative contribution of LHBS to corporate surplus would result in the opposite phenomenon, with growth in R3 and a reduction in R2. The resulting behavior is that of a “Success to the Successful” archetype between R2 and R3, where the growth of one feedback loop would drive continued growth at the expense of the other.

**Figure 3:**
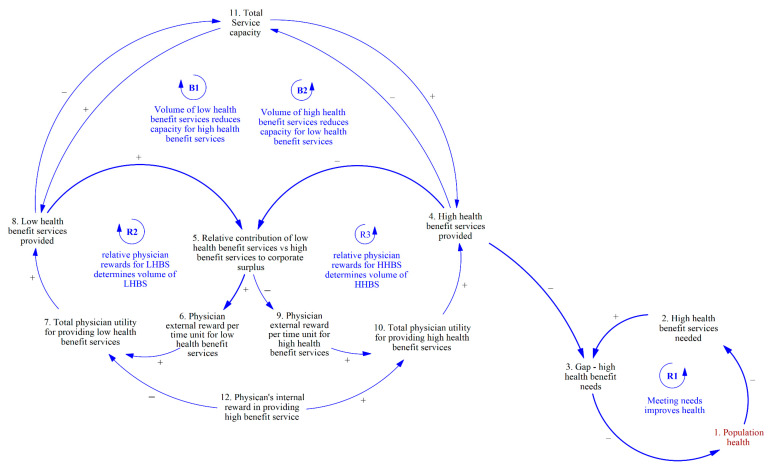
Levers influencing the mix between LHBS and HHBS are provided.

Figure 3 builds on Figure 2 with the addition of the levers that determine whether LHBS in R2 or HHBS in R3 are prioritized in a corporation. Firstly, an increase in total service capacity (#11) will result in the corporation’s capacity to produce a larger volume of both LHBS and HHBS but will not likely influence the mix between the two types of services. Balancing loops B1 and B2 describe how a limited total service capacity means that an increase in LHBS provided will limit the provision of HHBS (B1), and an increase in HHBS provided will limit the provision of LHBS (B2). Hence, this dynamic is described as a limit to growth between the two services. Secondly, a physician’s internal reward in providing a HHBS (#12) can result in an increase in the total utility of providing a HHBS (#10) but a lower total utility in producing a LHBS (#7). This internal reward may be described as satisfaction due to a sense of beneficence or justice in providing services that are of high benefit to their patients. However, this satisfaction may be limited by the knowledge of physicians as to the amount of health benefit each health service provides to their patients. The physician then determines the volume of LHBS or HHBS to provide by weighing their internal and external rewards.

**Figure 4:**
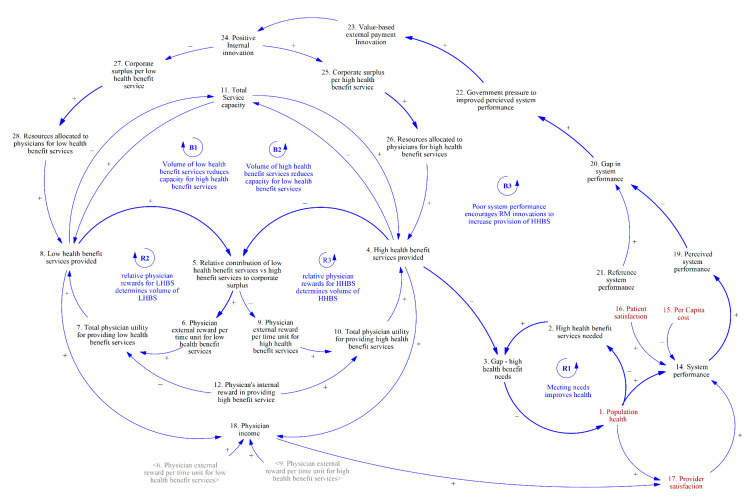
Government actions to increase the provision of HHBS to improve system performance.

In Figure 4, we describe how the mix of health services provided affect the overall health system performance and in turn, how the health system performance influences the government’s decisions to incorporate innovations in reimbursement mechanisms.

Balancing loop B3 describes how a decrease in system performance results in government action that pressures corporations to provide a higher volume of high-health benefit services and improve population health. As the dominant payor in Singapore and accountable to the general population, the government has a responsibility to influence the allocation of services to achieve a desirable mix of system performance metrics (#14) based on the quadruple aim outcomes: population health (#1), per capita cost (#15), patient satisfaction (#16), and provider satisfaction (#17). Provider satisfaction (#17) is determined mostly by physician income (#18), which is made up of the volume of services provided multiplied by the physician reward per unit of time for the services provided. The government conducts some measures of system performance according to its own metrics to provide a perceived view of system performance (#19). The gap (#20) between a reference system performance (#21), defined as the level of system performance the government wants to achieve, and the perceived system performance (#20), will manifest as some pressure for the government to act in order to improve system performance (#22). The government will do so by engaging in value-based external payment innovation (#23), which will place pressure on the corporation to innovate internally within the corporation (#24). Successful internal innovations aim to increase the corporate surplus from providing HHBS (#25) and thus the volume of HHBS provided (#26), while decreasing the corporate surplus from providing LHBS (#27) and the volume of LHBS provided (#28). These changes will alter the mix of services to increase the volume of HHBS provided (#4). Thus, these innovations will reduce the gap in high health benefit needs (#3), improve population health (#1), and improve system performance (#15) in the long run.

### 4.2. Relationship between RM Changes Considered and System Performance

Table 1 summarizes the six reinforcing loops R1, R2, and R3, and the balancing loops B1, B2, and B3. The loops R1, R2, and R3, as well as B1 and B2, show the general dynamics of retrospective payment-driven RMs (FFS and case-based payment), while the loop B3 shows the intended consequences of introducing RM reform through capitation. Note that all loops directly or indirectly affect system performance.

### 4.3. RM Innovation Strategies

In the CLD, the mechanism of RM innovation was not specified, other than that its effect is to influence the service mix on profit/surplus. Using the CLD, we discussed with our stakeholders how RM innovation can occur in the context of the Singapore health system.

At the outset, the stakeholders were asked to consider either variations in FFS or capitation as strategies to achieve improvements in the quadruple aim. As noted, in this simplified view, the provider organization will want to influence the service mix to the extent that a shift will lead to increased profit (or, for a not-for-profit organization, surpluses that offer the opportunity to grow, recruit the most talented staff, and achieve economies of scale). Under FFS, this creates an incentive for promoting services, irrespective of their benefit. As noted previously, under simple capitation, where payments to the organization are based purely on the number of individuals covered under the capitation arrangement (e.g., in Singapore, each RHS is associated with a defined geographical catchment area), the organization goal is the same, but the principal incentive is to minimize high-cost service utilization and to drive down the cost of services through increased efficiency.

One FFS-based RM innovation strategy discussed is to retain FFS but change the reimbursement structure to reflect the value of services (i.e., value-based pricing) [47,48]. However, changing the fee schedule based on value was noted to potentially involve an enormous list based on numerous patient features and a complex mix of service options, resulting in what is likely to be a counterproductive form of micromanagement requiring an expensive infrastructure.

In considering capitation strategies for RM innovation, it was appreciated that alone, capitation runs the risk of reducing services, irrespective of the health benefit or value [49,50]. To avoid the problem of underservice while improving all elements of the quadruple aim, basic capitation would need to be combined with some sort of reward or punishment based on the perceived performance gap. The effect is to level the playing field, as what had been high-margin LHBS are no longer rewarded, and what had been low-margin HHBS are no longer stifled. The overall effect would be to increase the proportion of HHBS as providers are no longer rewarded for high margin LBHS and to drive down the costs of these services through improved efficiencies.

It was noted that while the strategy of capitating with some adjustments based on perceived performance could address the problem of underservice, success depends on the ability to adequately measure the four cardinal features of system performance. The challenge is to accurately assess whether the aggregate of those four measures represents performance relative to expectations (the “performance gap”, #24). This measurement activity was noted to be a non-trivial task.

In addition, it was appreciated that capitating may also create stress among the providers since the capitated payment represents a “common pool” resource that is reallocated under a RM change in an effort to improve system performance. The reallocation can create “winners” and “losers”, as some providers gain resources at the expense of other providers.

## 5. Discussion

The developed CLD is a simple visual representation of the casual linkages between reimbursement mechanisms and behaviors of actors in the health system, the resulting mix of high and low health benefit services, and health system performance. It highlights how a volume-based system can lead not only to cost inflation but also incentivize high-margin services irrespective of their value. This system dysfunction is a manifestation of the dynamic structure of the system, represented by a series of feedback loops. The result of a weak linkage between the supply of services and the value of those services is that the distribution of low- and high-value services depends on the relative compensation for such services. The tendency to supply high-margin but low-value services is only counterbalanced by the providers intrinsic tendency to choose to provide high-value services without regard to financial or other reward.

In this context, capitation serves to alleviate the distribution of provided services based on margin, as reflected in loops B1a and B1b. The intended consequence of capitation is that it “fully aligns providers’ financial incentives to eliminate all major categories of waste” [26] and facilitates proactive management of high-cost patients [51]. In the CLD, the provision of low health benefit services might be seen as a form of waste if it means avoiding future, potentially higher costs. Under capitation, with providers being paid on a per-member, per-month basis, there are no perverse incentives to perform “shiny” services with high margins and high costs. However, the implementation of a capitation system linked to system performance presents operational challenges. These challenges relate to the difficulties of measuring system performance and governing common pool resources.

One observation from the CLD is that the proliferation of high-margin but low-value services can occur without requiring any malfeasance on the part of providers or service organizations. One reason is that reimbursement rates may depend on advocacy. As has been noted in the case of lumbar stenosis surgery, simpler interventions “usually have no sponsor beyond the conscientious and compassionate clinician” [52]. The other reason for the service-value mismatch is that the relative value of health services is poorly studied, poorly understood, and not integrated into the service fee structure. In the context of limited compelling scientific evidence and a disconnect between fees and health value, physicians are often operating in a “gray zone”. Both HHBS and LHBS are among the health services that are reasonable to provide to at least some patients, if only on the provider’s implicit belief that the patient receiving the service will have better health as a result, a belief reinforced by the system’s high reimbursement rate. Hence, we need positive, transformative change to realign reimbursement rates to health benefits.

### 5.1. Limitations

Our preliminary discussions with policy stakeholders show that the seed CLD has face validity and provides a framework to promote discussion about reimbursement changes in the Singapore public health system and potential desirable and undesirable effects on health system performance, particularly on population health and per capita cost. However, several limitations of the CLD became apparent. Importantly, the assumption that (1) organizations have well-defined mechanisms to set rewards per unit time for sets of services and that (2) providers make choices within a set of permissible services that range from low to high health benefits was deemed to be an oversimplification of the complicated set of motivators and factors at play in the real world. In the case of the public sector, where we focused our discussions, physician salaries are largely fixed, with variable components currently being geared towards volume and quality targets. At the administrative decision-making level, operations are governed by service level agreements, which include financial viability as well as patient experience measures such as waiting time and complaint rates.

When using the CLD to consider how reimbursement changes might affect system performance, it became apparent that the diagram does not account for potentially important secondary effects of capitation. In particular, in the discussions emerging from the CLD development, it became evident that a likely important secondary effect of capitation stress between providers was due to the reallocation of a common pool [26,53]. This problem has been discussed by Ostrom and others [54], with the potential to create a “tragedy of the commons” dynamic [55] in which providers may rapidly dry up the capitation common pool before the end of the payment cycle [56]. As she suggests in the domain of sharing natural resources, the challenge is to create a governance structure that creatively addresses the stresses between winners and losers. This could be relevant in the context of health care. Notably, such governance issues would involve each decision-making level (provider, RHS, and government) as well as between levels. For example, the providers could effectively work with the RHS to have the government increase the common pool by demonstrating improved system performance.

In addition, we also did not consider another form of capitation common in some countries but deemed not relevant to Singapore. In this variation, the organizations receive a capitated amount, typically based on historical reimbursement, but providers are paid based on FFS. Not only was the potential negative impact on system performance evident to the stakeholders, but it did not apply as providers in the Singapore public sector are primarily paid on a salary basis. Additionally, it was not seen as a solution relevant to the commissioning of private providers who might in the future be contracted to provide services under the public system.

### 5.2. Scope of Future Work

The CLD presented here is a basic, simple representation of the relationship between reimbursement mechanisms and health system performance. It will be revised based on further discussions with Singapore’s healthcare policy stakeholders as well as global experts. Apart from focusing on a more generalizable causal loop structure, we will also incorporate hypotheses regarding the secondary effects of capitation and the conditions that must apply for capitation to lead to sustained positive innovation in the health system. We suspect a major consideration of successful RM reform is the way in which the common pool under capitation is distributed, including how it is invested in efforts to develop, evaluate, and sustain innovations that promote whole-system performance. This issue has been described by Ostrom and others as the challenge of governing common pool resources [54,56].

### 5.3. Impact

Reform and innovation in RMs present challenges to policymakers because of the high stakes, the diversity of stakeholders, and the near certainty that the changes will engender secondary effects that could lead to policy failure. The introduction of capitation into a system that has historically been driven by retrospective payment methods calls for extensive engagement between stakeholders at different health system levels. A sustainable approach to capitation requires an evolution of mechanisms to guide the shared use of common pool resources between different actors in the health system. In this context, the present work provides a framework for more effective communication between stakeholders during the design and implementation of such reforms.

## 6. Conclusions

Our work presents a systems-level perspective on innovations in reimbursement mechanisms. The CLD developed highlights the feedback causal relations between the government, provider organizations, and physicians in determining the mix of health services provided in response to health system performance. In particular, our work accentuates the need for functional governance mechanisms at different levels of the health system to ensure that health services provided improve health system performance.

## Figures and Tables

**Table 1:** Summary of causal loops emerging from the Causal Loop Diagram.

Loop	Description	Variables
R1	Meeting needs improves health	1→2→3
R2	Relative physician rewards for LHBS determines the volume of LHBS	5→6→7→8
R3	Relative physician rewards for HHBS determines volume of HHBS	5→9→10→4→5
B1	Volume of low health benefit services reduces the capacity for high health benefit services	8→11→4→5→6→7→8
B2	Volume of high health benefit services reduces the capacity for low health benefit services	4→11→8→5→9→10→4
B3	Poor system performance encourages RM innovations to increase provision of high health benefit services	14→19→20→22→23→24→25→26→3→1→14

Causal Loop Diagram: CLD, LHBS: Low Health Benefit Services, HHBS: High Health Benefit Services, FFS: Fee for Service, RM: Reimbursement mechanisms.

## Data Availability

Models constructed can be made available upon reasonable request from the authors.

## Abbreviations

Causal Loop Diagram: CLD, LHBS: Low Health Benefit Services, HHBS: High Health Benefit Services, FFS: Fee for Service, RM: Reimbursement mechanisms.
